# DentFilm v1.0: an expert-annotated clinical image dataset of complete denture biofilm

**DOI:** 10.1016/j.dib.2026.113318

**Published:** 2026-10-08

**Authors:** Kelvin Vinícius Divino Rodrigues, Melissa Hollanda de Oliveira Alves, Thammyres Otto Barboza de Oliveira, Vitória Nogueira Oliveira, Pedro Henrique de Sousa Oliveira, Lara Kramer Chiomark Malaquias, Wander José da Silva, Luiz Eduardo da Silva, Raphael Cavalcante Costa

**Affiliations:** a School of Dentistry, Alfenas Federal University (UNIFAL-MG), Gabriel Monteiro da Silva 700, Alfenas, MG, 37130-001, Brazil; b Computer Science Department, Alfenas Federal University (UNIFAL-MG), Jovino Fernandes de Sales 2600, Alfenas, MG, 37133-840, Brazil; c Department of Prosthodontics and Periodontology, Piracicaba Dental School, Universidade Estadual de Campinas (UNICAMP), Piracicaba, SP, 13414-903, Brazil; d Department of Prosthodontics, School of Dentistry, Sāo Paulo University (USP), Sāo Paulo, SP, 05508-000, Brazil

**Keywords:** Denture biofilm, Dental plaque, Artificial intelligence, Computer vision, Image segmentation

## Abstract

Publicly available expert-annotated datasets are essential for developing robust artificial intelligence (AI) models in dentistry. Although several image datasets have been developed for applications involving natural teeth, no publicly available dataset currently exists for automated analysis of biofilm on conventional acrylic resin complete dentures. To address this gap, we present the first expert-annotated clinical image dataset specifically designed for computer vision research on complete denture biofilm. The dataset comprises 720 total files (630 standardized clinical photographs and 90 expert-annotated segmentation masks) obtained from 30 edentulous participants wearing maxillary conventional acrylic resin complete dentures. Images were acquired under controlled conditions at three stages of a denture hygiene protocol (before biofilm disclosure, after 0.5% basic fuchsin application, and after biofilm removal) and include frontal, right lateral, left lateral, right oblique, left oblique, occlusal, and intaglio views. Expert-generated pixel-level segmentation masks were manually created using the disclosed images as visual references, and each image is accompanied by structured metadata describing participant identification, acquisition stage, photographic view, and annotation files. The dataset is organized using a standardized directory structure to facilitate reproducible machine learning workflows and is publicly available through Zenodo. This resourceprovides the first publicly available benchmark dataset for complete denture biofilm analysis, supporting semantic segmentation, image classification, explainable AI, algorithm benchmarking, and the development of automated systems for denture hygiene assessment.

Specifications Table

**Table:** 

Subject	Dentistry; Artificial Intelligence; Computer Vision
Specific subject area	Prosthodontics; Complete denture biofilm; Medical image analysis
Type of data	Clinical images (.jpg), segmentation masks (.png), structured metadata (tabular format), documentation (.pdf/.txt)
Data collection	Standardized digital photographs of maxillary conventional acrylic resin complete dentures were acquired with a DSLR camera (Canon EOS Rebel T7) equipped with a 100-mm macro lens and a twin flash, using fixed settings (shutter speed 1/160s, aperture f/11, ISO 100, autofocus). Photographs were taken at three sequential stages of a denture-hygiene protocol (before biofilm disclosure, immediately after disclosure with a 0.5% basic fuchsin solution, and after complete biofilm removal) across seven standardized views (frontal, right/left oblique, right/left lateral, maxillary occlusal, and intaglio surface). Pixel-level annotations were manually generated by experienced prosthodontists using the disclosed images as reference.
Data format	Raw images, expert-annotated segmentation masks, and structured metadata
Data source location	School of Dentistry, Federal University of Alfenas (UNIFAL-MG), Alfenas, Minas Gerais, Brazil
Data accessibility	Repository name: Zenodo. Data identification number: DOI 10.5281/zenodo.22813487 Direct URL to data: https://doi.org/10.5281/zenodo.22813487
Related research article	None

## 1 Value of the Data

• DentFilm v1.0 is the first publicly available expert-annotated clinical image dataset dedicated to complete denture biofilm, addressing an important gap in artificial intelligence resources for removable prosthodontics. Existing publicly available dental image datasets are primarily focused on natural teeth and related oral conditions.
• The dataset comprises standardized clinical photographs of maxillary conventional acrylic resin complete dentures, acquired under controlled conditions at three stages of a denture hygiene protocol and from multiple standardized photographic views, ensuring consistency and reproducibility.
• Each image is accompanied by expert-generated pixel-level segmentation masks and structured metadata, enabling supervised machine learning applications, particularly semantic segmentation, image classification, and computer vision model development.
• The dataset provides a benchmark resource for developing, validating, and comparing automated methods for complete denture biofilm detection and quantification, facilitating reproducible research across different artificial intelligence algorithms.
• DentFilm v1.0 may benefit researchers in prosthodontics, dentistry, computer vision, biomedical engineering, and artificial intelligence, supporting the development of automated tools for denture hygiene assessment, explainable AI, and future multimodal learning approaches.

## 2 Background

Complete edentulism affects approximately 350–400 million people worldwide, with this number expected to increase due to population aging [1]. Conventional complete dentures made of acrylic resin are widely used for oral rehabilitation [2]; however, their surfaces favor biofilm accumulation, which is associated with denture stomatitis and systemic complications, such as aspiration pneumonia in frail older adults [3]. Denture hygiene may be compromised by reduced manual dexterity and motor limitations, making biofilm monitoring relevant for the prevention and control of these conditions.

Conventional biofilm assessment relies on disclosing solutions followed by visual inspection or computerized analysis [[4], [5], [6], [7], [8]]. Although useful, these methods are time-consuming, require trained examiners, and may present variability. Advances in artificial intelligence (AI) enable automated analyses of dental images [[9], [10], [11], [12], [13]], but depend on standardized and annotated datasets. However, no public dataset specifically addressing biofilm on complete dentures was previously available, despite their distinct characteristics compared with natural teeth [14]. Therefore, DentFilm v1.0 was developed, comprising standardized photographs of maxillary complete dentures acquired before disclosure, after disclosure, and after biofilm removal. Disclosed images were used to generate pixel-level segmentation masks, while corresponding undisclosed images were preserved for computer vision applications.

## 3 Data Description

### 3.1 Dataset overview

DentFilm v1.0 comprises standardized clinical photographs, expert-generated binary segmentation masks, structured metadata, and supporting documentation organized in a publicly accessible Zenodo repository (DOI: 10.5281/zenodo.22813487). The repository was designed to facilitate data reuse by providing standardized file organization, image–mask correspondence, and comprehensive metadata describing each image according to acquisition stage and photographic view.

### 3.2 Dataset composition

DentFilm v1.0 comprises 720 total files (630 standardized clinical photographs and 90 expert-annotated segmentation masks) of maxillary conventional acrylic resin complete dentures obtained from 30 complete-denture wearers under controlled clinical conditions. For each participant, images were acquired at three sequential stages of the denture hygiene protocol: before biofilm disclosure, after biofilm disclosure with 0.5% basic fuchsin disclosing solution, and after complete biofilm removal. At each stage, seven standardized photographic views were obtained, including frontal (0°), right and left oblique (45°), right and left lateral (90°), maxillary occlusal, and denture intaglio surface. To support the development of AI models for biofilm detection from unstained clinical photographs, images acquired before biofilm disclosure serve as model inputs, whereas the corresponding expert annotations constitute the reference standard (ground truth) (Table 1).

**Table 1:** Composition and organization of the dataset.

Dataset component	Participants (*n*)	Clinical stages (*n*)	Standardized views (*n*)	Calculation	Total files (*n*)
Clinical photographs	30	3	7	30 × 3 × 7	630
Binary annotation masks	30	—	3 Annotated views per participant	30 × 3	90
**Total dataset**	**30**	**3**	**—**	**630** **+** **90**	**720**

Note. The dataset comprises 630 clinical photographs and 90 manually annotated binary masks for biofilm segmentation, resulting in 720 files in total.

### 3.3 Clinical image dataset

The clinical image dataset consists of standardized digital photographs acquired under controlled conditions using a predefined imaging protocol. Images were captured in JPEG format with a native resolution of 6000 × 4000 pixels and 24-bit RGB color depth using standardized camera settings, illumination, background, and participant positioning to minimize technical variability and ensure image consistency across participants. Prior to computational analysis, all images were resized to 240 × 160 pixels while preserving their original aspect ratio, reducing computational cost without introducing geometric distortion. Only photographs meeting predefined quality criteria for focus, exposure, illumination, framing, and complete visualization of the denture surface were included in the final dataset.

### 3.4 Annotation dataset

Pixel-level annotations were generated to provide the reference standard for the development and evaluation of AI models for complete denture biofilm segmentation. The disclosed images served as the visual reference for manual delineation of biofilm-covered areas by expert examiners. The resulting binary segmentation masks were paired with the corresponding photographs acquired before biofilm disclosure, enabling supervised learning from unstained clinical images while preserving clinically validated ground-truth annotations. These masks provide reference labels for semantic segmentation, image classification, and quantitative biofilm analysis.

### 3.5 Metadata structure

The DentFilm v1.0 repository includes structured metadata describing each clinical image and its corresponding annotation. Metadata are provided in tabular format and include an anonymous participant identifier, acquisition stage, photographic view, image filename, corresponding segmentation mask filename, and dataset partition, when applicable. Demographic information, including participant age and sex, is also provided in an Excel spreadsheet in the Zenodo repository to further characterize the study population while preserving participant anonymity. The 30 participants had a mean age of 64.1 ± 8.7 years (range 49–82 years) and comprised 20 males (66.7%) and 10 females (33.3%). This structure enables automatic image-mask pairing, ensures traceability, and facilitates integration into machine learning and computer vision workflows.

### 3.6 Repository organization

The DentFilm v1.0 repository follows a hierarchical directory structure designed to facilitate data access, interoperability, and reproducibility. Clinical photographs, segmentation masks, metadata, and supporting documentation are organized into separate directories using standardized filenames and metadata records to ensure consistent correspondence between images and annotations. This organization is compatible with commonly used computer vision and semantic segmentation frameworks, facilitating the implementation of custom training, validation, and testing pipelines. The complete repository organization is illustrated in Fig. 1.

**Fig. 1:**
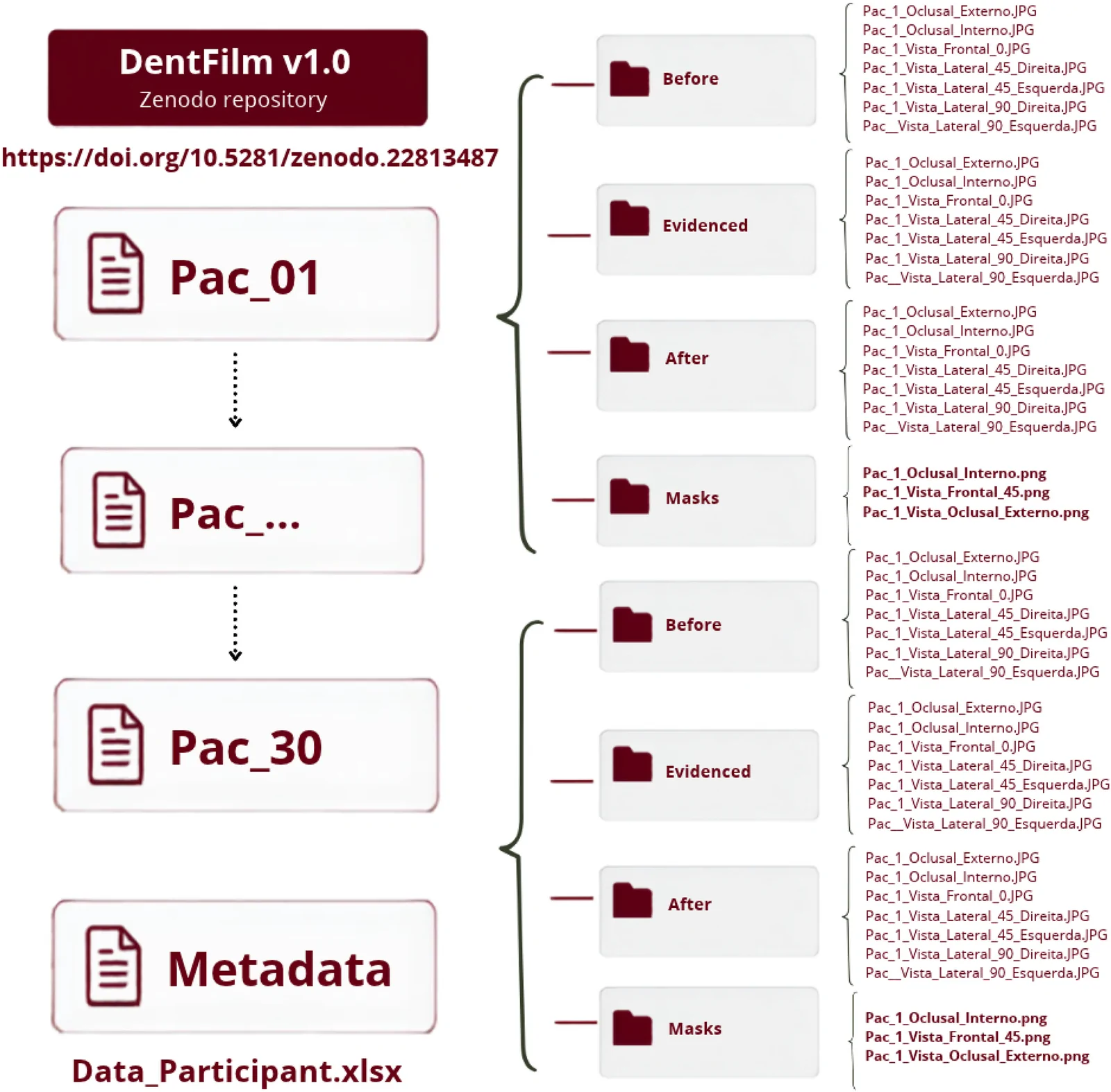
Organization of the DentFilm v1.0 repository. Clinical photographs, segmentation masks, metadata, and documentation are organized into separate directories. The metadata file establishes the correspondence between each clinical image and its respective segmentation mask through standardized filenames and participant identifiers, facilitating automated data loading and integration into computer vision workflows. The dataset is publicly available through the Zenodo repository (DOI: 10.5281/zenodo.22813487). The graphical layout 1 was created with assistance from ChatGPT (OpenAI) based on the authors' conceptual design and subsequently reviewed, edited, and approved by the authors.

## 4 Experimental Design, Materials and Methods

### 4.1 Study design and ethical approval

DentFilm v1.0 was retrospectively assembled from standardized clinical photographs acquired of the School of Dentistry, Federal University of Alfenas (UNIFAL-MG), Brazil, between April 2025 and July 2026. The study protocol was approved by the local Research Ethics Committee (No. 96996226.8.0000.5142), and all participants provided written informed consent before enrollment. Fig. 2 summarizes the complete workflow used to construct DentFilm v1.0, including participant recruitment, clinical image acquisition, image annotation, metadata generation, and repository organization.

**Fig. 2:**
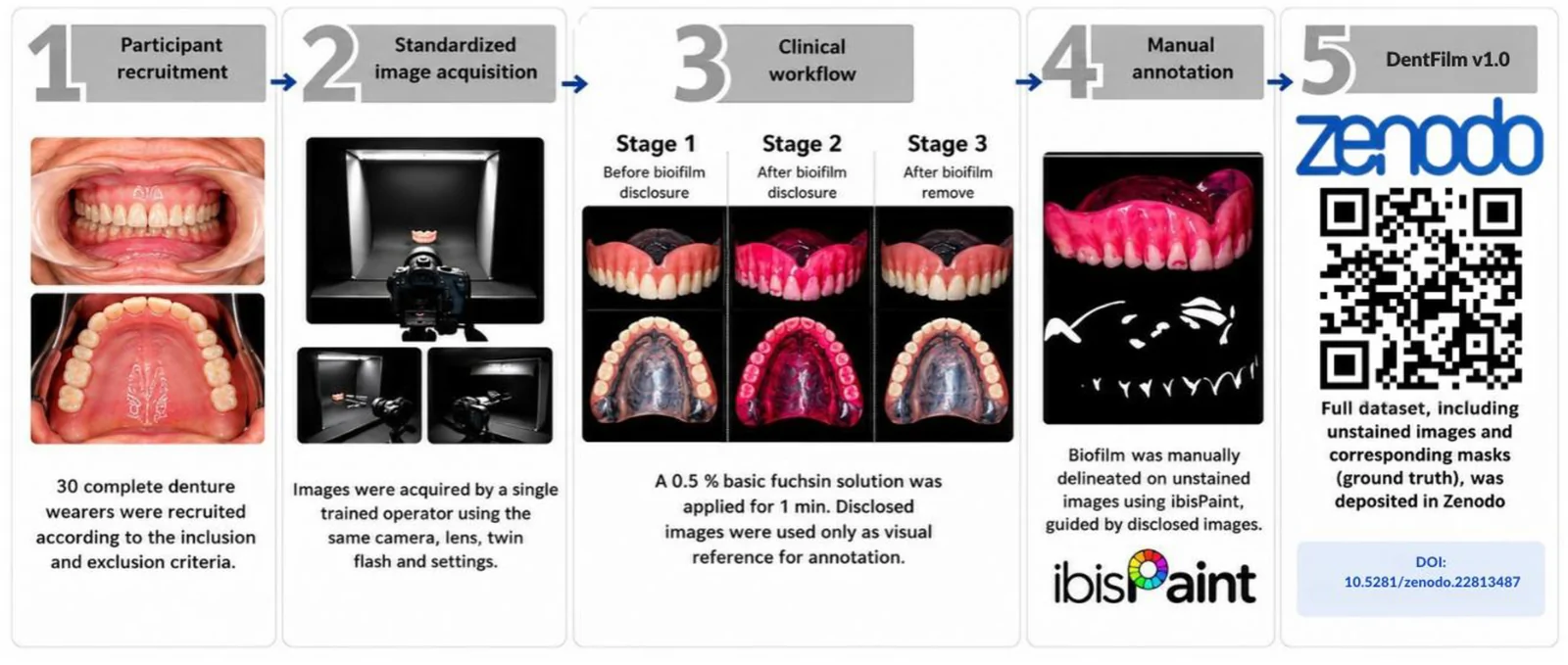
Workflow for the construction of the DentFilm v1.0 dataset. Standardized clinical photographs were acquired from complete-denture wearers under controlled conditions following participant recruitment and informed consent. For each participant, images were obtained at three sequential clinical stages: before biofilm disclosure, after disclosure with 0.5% basic fuchsin, and after complete biofilm removal. The disclosed photographs were used exclusively as visual references for manual pixel-level annotation of the corresponding unstained images, generating binary segmentation masks (ground truth). The final repository comprises standardized clinical images, expert-generated annotation masks, structured metadata, and supporting documentation, and is publicly available through the Zenodo repository (DOI: 10.5281/zenodo.22813487).

### 4.2 Participant recruitment

Participants were recruited from patients attending the Dental School Clinic of the UNIFAL-MG. Inclusion criteria comprised complete edentulism, good general health, and the use of maxillary complete dentures with adequate adaptation and retention according to the criteria proposed by Kapur [15] assessed by a single examiner (K.V.D.R). Exclusion criteria included denture-related inflammation of the oral tissues (i.e.; denture stomatitis), systemic or local conditions predisposing to *Candida* spp. infection, use of antibiotics or antifungal medications within three months before or during the study, and cognitive impairment, defined as a Mini-Mental State Examination (MMSE) score below 13 [16]. These eligibility criteria were established to ensure standardized image acquisition and improve the consistency and reliability of the resulting dataset. All participants were evaluated regarding the inclusion and exclusion criteria, including the Kapur index. However, all of them, including those whose dentures were classified as inadequate according to the Kapur criteria, were considered for image acquisition and dataset expansion, as a simulation of the real clinical condition of these individuals.

### 4.3 Conventional complete denture characteristics

Only functional maxillary conventional complete dentures in clinical use were included. All dentures presented the conventional design consisting of acrylic resin artificial teeth and a pink heat-polymerized polymethyl methacrylate (PMMA) denture base with complete palatal coverage, which represents the most common configuration used in routine clinical practice.

### 4.4 Biofilm disclosure protocol

Pilot tests were conducted to standardize the biofilm disclosure procedure and identify the conditions providing adequate staining contrast, homogeneity, and reproducibility across samples.For the standardized protocol, each denture was fully immersed in a 0.5% basic fuchsin solution (Quimisul SC, Brazil) for 1 min, followed by rinsing under running water for 10 s to remove excess dye. This protocol was selected based on the pilot evaluation because it provided uniform staining, adequate visual contrast, and reproducible biofilm disclosure.

Clinical photographs were obtained at three sequential stages: before biofilm disclosure, immediately after disclosure, and after complete biofilm removal. Following the second acquisition, dentures were thoroughly cleaned under running water with neutral soap, and residual biofilm and staining were removed by professional mechanical polishing using acrylic polishing burs until no visible biofilm or staining remained. A final set of standardized photographs was then acquired.The disclosed photographs were used exclusively as visual references for manual annotation and were not used as input images for artificial intelligence model training.

### 4.5 Standardized clinical image acquisition

Standardized digital photographs of the maxillary complete dentures were acquired using a digital DSLR camera (Canon EOS Rebel T7, Canon Inc., Tokyo, Japan) equipped with a 100-mm macro lens (Canon EF 100 mm f/2.8 Macro) and a twin flash (Macro Twin Lite MR-14EX II, Canon Hong Kong Co., Ltd.). All photographs were obtained by a single operator (K.V.D.R) using identical camera settings: shutter speed 1/160 s, aperture f/11, ISO 100, and autofocus [17]. Images were stored in JPEG format with a resolution of 6000 × 4000 pixels and 24-bit color depth.

For each participant, seven standardized views were acquired at each clinical stage: frontal (0°), right and left oblique (45°), right and left lateral (90°), maxillary occlusal, and denture intaglio surface. For AI model development, three representative images per participant were selected one frontal (0°), one oblique (45°), and one lateral (90°) view because these views provide comprehensive visualization of the denture surfaces most frequently affected by biofilm accumulation while substantially reducing the time required for pixel-level annotation. Fig. 3 illustrates the standardized photographic protocol, showing the seven denture views captured at each of the three biofilm-disclosure stages.

**Fig. 3:**
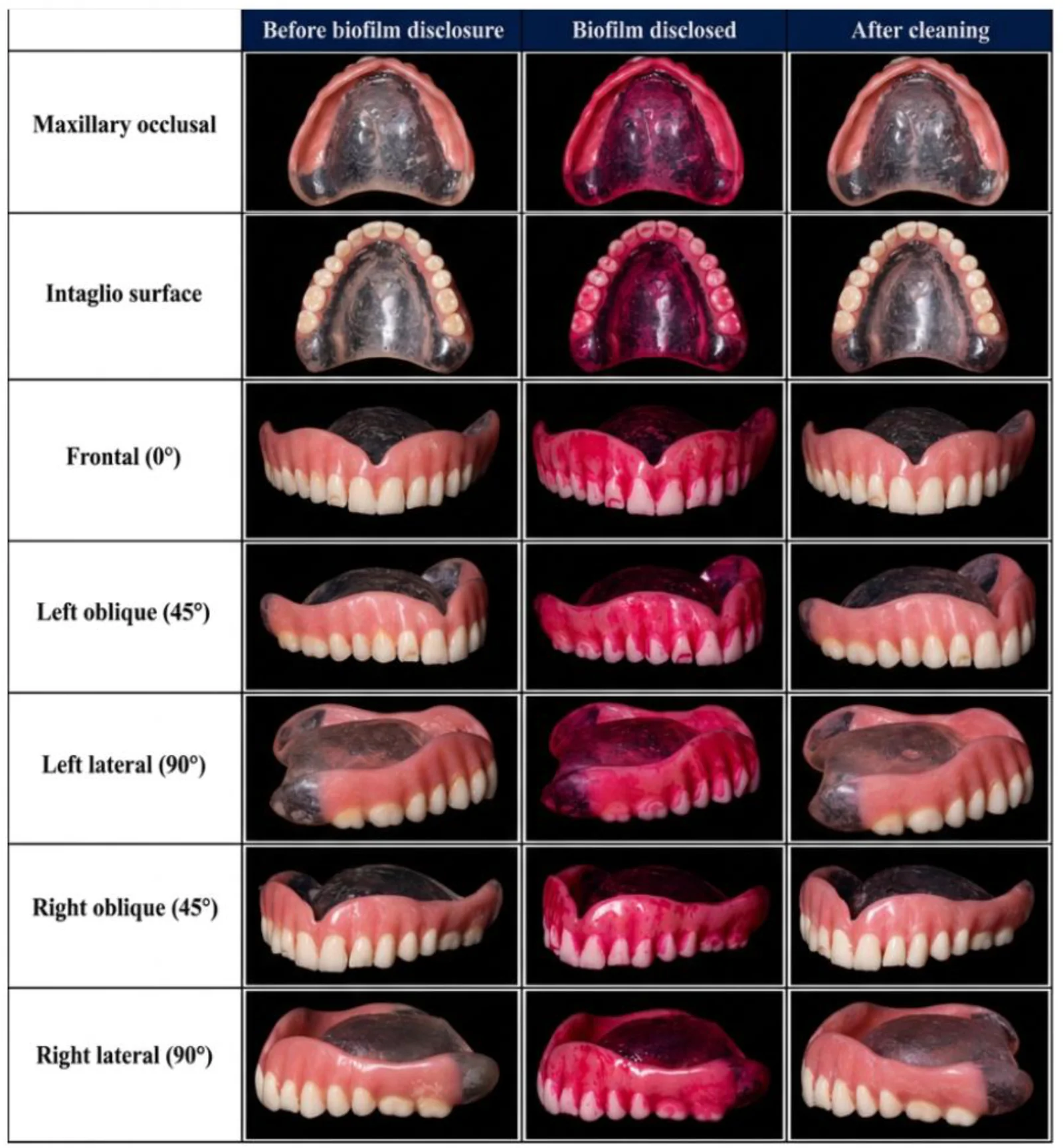
Standardized photographic protocol used to construct the DentFilm v1.0 dataset. Representative images showing the seven standardized views obtained for each maxillary complete denture at three sequential clinical stages: before biofilm disclosure, after disclosure with 0.5% basic fuchsin, and after complete biofilm removal. These standardized views ensured consistent image acquisition throughout the dataset and served as the basis for subsequent image selection and annotation.

### 4.6 Image quality control and preprocessing

All clinical photographs were visually inspected before inclusion to verify image quality and adherence to the standardized acquisition protocol. Images presenting inadequate focus, lighting artifacts, motion blur, incomplete visualization of the denture surface, or deviations from the acquisition protocol were excluded (*n* = 60) during the initial quality-control screening, before inclusion in the dataset. Following this screening, 630 standardized clinical photographs were retained for the final dataset, corresponding to 30 participants × 3 clinical stages × 7 standardized views, consistent with the dataset composition described in Section 3.2.

### 4.7 Image annotation protocol

Reference segmentation masks (ground truth) were manually generated by one trained examiner (T.O.B.O.) using the ibisPaintX application (ibis Inc., Tokyo, Japan). Manual delineation was performed on the unstained clinical photographs while the corresponding biofilm-disclosed image of the same denture was simultaneously displayed as a visual reference to accurately identify biofilm boundaries. This approach was adopted because the photographs acquired before and after biofilm disclosure presented slight differences in denture positioning, preventing precise pixel-wise spatial registration between the two images. Therefore, the disclosed photographs were used exclusively to guide the annotation process and were never included in the released dataset or used as input images for AI model training. The workflow used to generate the reference segmentation masks is illustrated in Fig. 4.

**Fig. 4:**
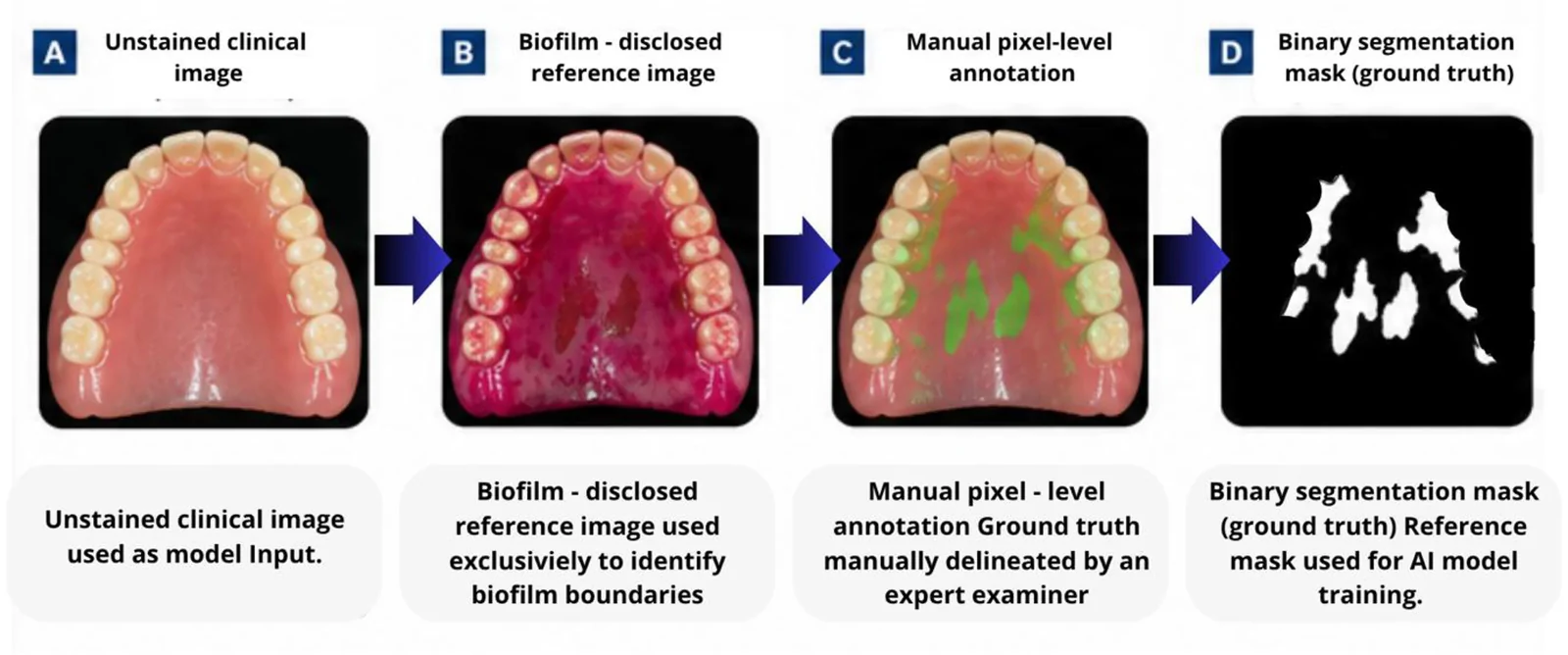
Workflow for generating the reference segmentation masks (ground truth). The annotation process consisted of four sequential steps: (A) acquisition of the unstained clinical photograph, which served as the input image; (B) acquisition of the corresponding biofilm-disclosed photograph, used exclusively as a visual reference to identify biofilm boundaries; (C) manual pixel-level delineation of the biofilm on the unstained image by a trained examiner; and (D) generation of the corresponding binary segmentation mask (ground truth) used for AI model development. The biofilm-disclosed photographs were not included in the released dataset and were never used as input images for model training. White = Biofilm and Black = Background.

### 4.8 Metadata collection and data anonymization

For each clinical image, metadata describing the participant identification code, acquisition stage (before biofilm disclosure, after biofilm disclosure, or after complete biofilm removal), photographic view (frontal, oblique, lateral, occlusal, or intaglio), and the corresponding manual segmentation mask were recorded to facilitate dataset organization and AI model development. Before repository organization, all data were anonymized by removing participant-identifiable information and replacing personal identifiers with unique participant codes. These identifiers were consistently applied across clinical images, segmentation masks, and metadata to ensure accurate correspondence while preserving participant confidentiality. The released dataset contains only de-identified data and complies with the approved ethical protocol and informed consent for public data sharing.

## Limitations

Images were acquired at a single clinical center using one imaging system and include only maxillary complete dentures; although acquisition was highly standardized, the current dataset does not encompass variations associated with different cameras, illumination systems, clinical environments, or prosthesis types. Manual segmentation was performed by a single trained examiner and represents an approximate delineation of the biofilm region rather than a histological ground truth. The current sample of 30 participants also limits the diversity and external generalizability of models developed exclusively from this dataset. Nevertheless, the dataset represents a specialized clinical resource comprising standardized clinical images and expert-generated pixel-level annotations, whose acquisition requires controlled clinical procedures, rigorous quality control, and specialist annotation, making the generation of such resources particularly challenging in health-related artificial intelligence research. DentFilm v1.0 should therefore be interpreted as an expandable initial release rather than a definitive and closed dataset. Future versions are expected to incorporate larger numbers of independent participants, multicenter acquisitions, multiple expert annotations, additional prosthesis categories, and complementary imaging modalities, including 3D scanning and smartphone-acquired images, to increase dataset diversity and representativeness. Data augmentation was not employed to compensate for the limited number of participants, as synthetically derived images do not replace independent clinical samples and may increase the risk of overfitting if inappropriately applied. The acquisition of additional independent clinical data is therefore considered the appropriate strategy for strengthening dataset diversity and model generalizability.

## Ethics Statement

The study was approved by the Research Ethics Committee of the Federal University of Alfenas (UNIFAL-MG), Brazil (CAAE No. 96996226.8.0000.5142). All participants provided written informed consent prior to enrollment and for the use of their data for research purposes. The study was conducted in accordance with the principles of the Declaration of Helsinki.

## CRediT Authorship Contribution Statement

Kelvin Vinicius Divino Rodrigues: Conceptualization, Methodology, Investigation, Resources, Writing- Original Draft, Writing – Review & Editing, Visualization. Pedro Henrique de Sousa Oliveira: Writing – review & editing, Methodology. Melissa Hollanda de Oliveira Alves: Software, Writing – review & editing, Software. Vitória Nogueira Oliveira: Writing – review & editing, Methodology. Thammyres Otto Barboza de Oliveira: Writing – review & editing, Methodology. Lara Kramer Chiomark Malaquias: Writing – review & editing, Methodology. Wander José da Silva: Writing – review & editing. Luiz Eduardo da Silva: Software, Supervision, Writing – review & editing, Writing – original draft. Accountability for all aspects of the work. Raphael Cavalcante Costa: Writing – original draft, Writing – review & editing, Supervision. All authors actively participated in discussing the manuscript findings and revised and approved the final version of Declaration of Competing Interest.

## Data Availability

The dataset supporting the findings of this study is publicly available in the Zenodo repository underDOI: 10.5281/zenodo.22813487(https://doi.org/10.5281/zenodo.22813487).The repository includes the original clinical photographs, manually annotated segmentation masks, complete structured metadata, annotation files, documentation, and the recommended directory organization required to facilitate data reuse and reproducibility. These materials are provided to support transparency, validation of the reported results, and future research on artificial intelligence–based segmentation of denture biofilm.

## Declaration of generative AI

Artificial intelligence (AI) tools were used exclusively to assist with English language editing, grammar correction, and improvement of writing fluency. The study design, methodology, data analysis, interpretation of the results, and all scientific content were conceived and prepared by the authors, who assume full responsibility for the final manuscript.
